# Food group intakes and high-sensitivity C-reactive protein among community-dwelling Japanese adults: a cross-sectional study

**DOI:** 10.1017/S1368980024001599

**Published:** 2024-10-18

**Authors:** Takashi Matsunaga, Kenji Wakai, Nahomi Imaeda, Chiho Goto, Yudai Tamada, Yasufumi Kato, Yoko Kubo, Rieko Okada, Mako Nagayoshi, Takashi Tamura, Asahi Hishida, Hiroaki Ikezaki, Jun Otonari, Naoyuki Takashima, Naoko Miyagawa, Keitaro Matsuo, Japan Multi-Institutional Collaborative Cohort (J-MICC) Study Group

**Affiliations:** 1 Department of Preventive Medicine, Nagoya University Graduate School of Medicine, Nagoya, Aichi 466-8550, Japan; 2 Department of Nutrition, Faculty of Wellness, Shigakkan University, Obu, Aichi, Japan; 3 Department of Public Health, Nagoya City University Graduate School of Medical Sciences, Nagoya, Aichi, Japan; 4 Department of Health and Nutrition, School of Health and Human Life, Nagoya Bunri University, Nagoya, Aichi, Japan; 5 Department of International and Community Oral Health, Tohoku University Graduate School of Dentistry, Sendai, Miyagi, Japan; 6 Department of Comprehensive General Internal Medicine, Faculty of Medical Sciences, Kyushu University, Fukuoka, Japan; 7 Department of General Internal Medicine, Kyushu University Hospital, Fukuoka, Japan; 8 Department of Psychosomatic Medicine, Graduate School of Medical Sciences, Kyushu University, Fukuoka, Japan; 9 Department of Public Health, Faculty of Medicine, Kindai University, Osaka-Sayama, Osaka, Japan; 10 Department of Public Health, Shiga University of Medical Science, Otsu, Shiga, Japan; 11 Department of Preventive Medicine and Public Health, Keio University School of Medicine, Tokyo, Japan; 12 Division of Cancer Epidemiology and Prevention, Aichi Cancer Center Research Institute, Nagoya, Aichi, Japan.; 13 Department of Cancer Epidemiology, Nagoya University Graduate School of Medicine, Nagoya, Aichi, Japan

**Keywords:** Food group, High-sensitivity C-reactive protein, Chronic inflammation, Cross-sectional study, Community-dwelling adults

## Abstract

**Objective:**

Healthy dietary patterns have been linked to lower levels of chronic inflammation. The present study aimed to investigate the associations between food group intakes and high-sensitivity C-reactive protein (hsCRP) among community-dwelling adults.

**Design:**

Cross-sectional.

**Setting:**

Three areas in Japan (Shiga, Fukuoka, or Kyushu and Okinawa).

**Participants:**

The present analysis included 13 648 participants (5126 males and 8522 females; age range, 35–69 years) who had been enrolled in the baseline survey of the Japan Multi-Institutional Collaborative Cohort Study. Food group intakes were estimated using a FFQ. Multiple linear regression was used to examine associations between the quartiles of each energy-adjusted food group intake and log-transformed hsCRP.

**Results:**

The following concentration ratios of hsCRP after comparing the highest and lowest quartiles of food group intake were significant: in males, 1·12 (95 % CI 1·02, 1·22) for processed meat, 1·13 (95 % CI 1·03, 1·24) for fish and 0·83 (95 % CI 0·76, 0·90) for nuts; in females, 0·89 (95 % CI 0·81, 0·97) for bread, 1·11 (95 % CI 1·03, 1·19) for processed meat, 0·86 (95 % CI 0·80, 0·92) for vegetables, 1·19 (95 % CI 1·11, 1·29) for fruit, 0·90 (95 % CI 0·84, 0·97) for nuts and 0·88 (95 % CI 0·82, 0·95) for green tea.

**Conclusions:**

Processed meat and nut intakes were associated with higher and lower hsCRP levels, respectively, in both sexes. However, for several food groups, including fish and fruit, previous findings from dietary pattern analyses were not supported by the present analyses at the food group level.

Inflammation plays a vital role in protecting the host from toxins and infection and resolves once these insults have been removed. However, under the presence of triggers, including chronic infection, unhealthy lifestyles and adverse psychological states, an acute inflammatory response can develop into systemic chronic inflammation, which causes tissue degeneration and diseases^(1)^. Experimental and observational studies have shown that chronic inflammation can increase the risks of diabetes, CVD, cancers, sarcopenia, frailty and all-cause mortality^(2)^.

Healthy dietary patterns, including the Mediterranean diet and Dietary Approaches to Stop Hypertension (DASH), have been associated with lower concentrations of C-reactive protein (CRP) based on the results of systematic reviews and meta-analyses of randomised controlled trials (RCT)^(3,4)^. These dietary patterns have common features: higher intakes of fruit, vegetables, fish, nuts and whole grain combined with lower intakes of red meat, processed foods and refined grains. However, some issues should be resolved for dietary recommendations. First, less remains known about the effects of individual food groups on chronic inflammation, and the results of previous studies examining the effects of individual food groups do not necessarily agree with those of dietary pattern analyses^(5,6)^. Because food group intakes are often correlated with each other, food groups suggested to affect inflammatory biomarkers by dietary pattern analyses may have inconsistent effects in isolation^(7)^. These inconsistent isolated effects with dietary pattern analyses may arise when the findings of dietary pattern analyses examined in one population are applied to another population with a different dietary pattern from the former. Second, relatively few studies have examined the associations between individual food groups and chronic inflammation in Asian populations^(8,9)^. Dietary patterns in Japan (e.g. high consumption of white rice and green tea) are distinct from those in other countries^(10)^. Therefore, more food group analyses should be conducted to clarify the roles of individual food groups on chronic inflammation in the Japanese population.

Therefore, the present cross-sectional study aimed to investigate the associations between individual food groups and high-sensitivity C-reactive protein (hsCRP) among community-dwelling Japanese adults.

## Methods

## Study design and participants

The present cross-sectional study was based on baseline data from the Japan Multi-Institutional Collaborative Cohort (J-MICC) Study. The J-MICC Study is an ongoing prospective cohort study aiming to detect gene–environment interactions that can lead to lifestyle-related diseases, including cancers^(11)^.

**Fig. 1:**
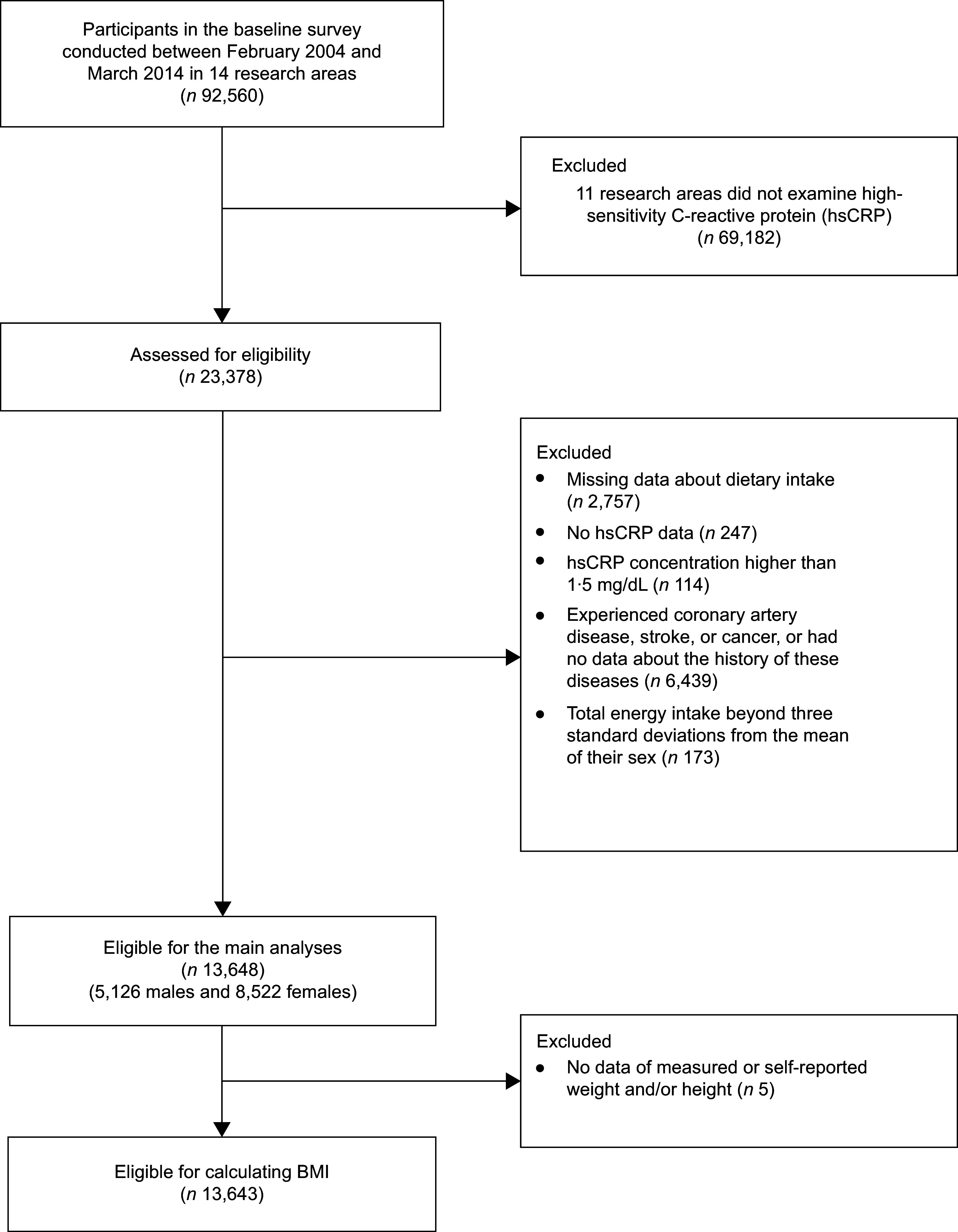
Flow diagram showing the selection of eligible participants

From February 2004 through March 2014, baseline surveys of the J-MICC Study were conducted in fourteen research areas of Japan. The participants were aged 35–69 years at the baseline surveys and recruited from community inhabitants, employees of companies or local governments, health checkup examinees, and first-visit patients at a cancer hospital. All participants completed a self-administered lifestyle-related questionnaire including items on food group intakes and medical history. Height and weight were measured at the health checkups. A total of 92 560 participants were recruited. In three areas (Shiga, Fukuoka, Kyushu or Okinawa), where 23 378 participants were enrolled, hsCRP was measured. Among these participants, the present study further excluded 2757 individuals who had missing data in at least one item necessary to calculate food group intakes, 247 those who had no data on hsCRP, 114 those who had an hsCRP concentration higher than 1·5 mg/dl, 6439 those who had a history of coronary artery disease, stroke, or cancer, or no data about the history of these diseases and 173 those whose total energy intake was beyond 3 sd from the mean of their sex (males: 742–3269 kcal/d, females: 780–2378 kcal/d). Therefore, a total of 13 648 participants (5126 males and 8522 females) were included in the present analysis (Fig. 1). Individuals with an hsCRP concentration higher than 1·5 mg/dl or co-morbidities were excluded because individuals with diseases that elevate hsCRP levels may have modified their dietary habits after the diseases had developed (reverse causation). Regarding the cut-off of hsCRP concentrations, a review article has reported that hsCRP concentration of healthy individuals is usually lower than 1·0 mg/dl^(12)^. Additionally, a meta-analysis has referred to CRP concentrations of > 0·3 mg/dl as high levels and reported that the relative risk of CVD mortality was about 4·0 at 0·9 mg/dl compared with < 0·1 mg/dl. Therefore, we regarded at least hsCRP levels higher than 1·5 mg/dl as pathological^(13)^. The dataset version 200819 was used in the present analysis.

## Exposure

Food group intakes were evaluated using a forty-seven-item FFQ^(14)^ and independent items for beverages. The FFQ included items regarding how often the participants had consumed forty-six foods and non-alcoholic beverages during the previous year. The frequencies of eating rice, bread and noodles at breakfast, lunch and dinner were categorised into six levels, from rarely to every day. For these staple foods, the participants were also asked about portion sizes. Regarding the other forty-three foods and beverages, their frequencies were categorised into eight levels, from rarely to three times/d or more, and their portion sizes were predefined. For alcoholic beverages, their frequencies were categorised into six levels, from rarely to seven times per week, and their portion sizes were also queried. In the present analysis, the following food groups were considered as exposures: rice (one item), bread (one item), red meat (one item: beef/pork), processed meat (one item: ham/sausage/bacon), poultry (one item: chicken), dairy products (three items: butter, milk and yogurt), fish (three items: fish, bone-edible small fish and canned tuna), vegetables (ten items: pumpkin, carrots, broccoli, green-leaf vegetables, other green-yellow vegetables, cabbage, radishes, dried radishes, burdock/bamboo shoots and other vegetables), fruit (two items: citrus fruits and other fruits), nuts (one item: peanuts/almonds), coffee (one item: regular/instant coffee) and green tea (one item). These food groups or the nutrients they contain have been reported to be associated with chronic inflammation^(3–6,15)^.

A validation study compared intakes of total energy and twenty-six nutrients estimated using the FFQ with those using a 3-d weighed diet record^(16)^. Log-transformed, energy-adjusted and de-attenuated correlation coefficients for total energy intake and twenty-six nutrients ranged from 0·12 to 0·86 in males and from 0·10 to 0·66 in females. The FFQ also showed high 1-year interval reproducibility for the intakes of total energy, nutrients and food groups^(17)^. Furthermore, another validation study compared food group intakes using the FFQ with those using four 3-d weighed diet records^(18)^. The energy-adjusted and de-attenuated correlation coefficients between the second FFQ and four 3-d weighed diet records were as follows in males and females, respectively: 0·63 and 0·66 for rice, 0·75 and 0·70 for bread, 0·42 and 0·43 for meat, 0·81 and 0·54 for dairy products, 0·61 and 0·69 for fish, 0·35 and 0·49 for green vegetables, 0·30 and 0·24 for other vegetables, 0·69 and 0·48 for fruit, 0·60 and 0·56 for coffee, and 0·64 and 0·69 for green tea^(18)^.

In addition to the FFQ, our questionnaire also included items evaluating coffee and green tea consumption. The present analyses used coffee and green tea intakes estimated by these items because they could evaluate a higher amount of drinking beverages than could the FFQ (the amount of coffee and green tea consumption ranged from rarely to five cups/d or more and from rarely to ten cups/d or more, respectively).

## Outcomes

At the health checkups, blood samples were drawn in a 7-ml tube for serum and a 7-ml EDTA-2Na-containing tube for plasma and buffy coat. In the Shiga area, serum hsCRP was measured by the latex agglutination method using an N-assay LA CRP-S reagent (Nittobo Medical) and a JCA-BM8060 analyzer (JEOL Ltd). In the Fukuoka and Kyushu or Okinawa areas, the same measurement was conducted by the nephelometry method using an N-latex CRP II reagent (Siemens Healthcare Diagnostics Ltd) and a Behring Nephelometer II analyzer (Siemens Healthcare Diagnostics Ltd). Linear regression showed good agreement between the two measurement methods (Y = 1·0122X + 0·0027, R^2^ = 0·99 006), where X are hsCRP concentrations measured by the method in the Shiga area and Y are those measured by the method in the Fukuoka and the Kyushu or Okinawa areas.

## Covariates

When assessing the associations between food group intakes and hsCRP, the following demographic, lifestyle and dietary factors were adjusted as covariates: age, total energy intake, residential area, educational attainment, smoking exposure, alcohol consumption and total physical activity. These factors have been reported to be associated with chronic inflammation and/or adjusted in observational studies that examined the associations between food group intakes and inflammatory markers^(1,6,19)^. In addition to the residual method, total energy intake was incorporated into covariates. This adjustment may reduce random error if total energy intake is associated with hsCRP independent of food group intakes^(20)^. The following continuous variables were converted into categorical variables because possible non-linear associations between these variables and hsCRP should be considered: age, total energy intake, smoking exposure, alcohol consumption and total physical activity. Cut-off values of these categorical variables are presented in Table 1 by sex.

**Table 1:** Characteristics of participants according to hsCRP quartiles by sex

Males									
Characteristics	Quartiles of hsCRP (mg/dl)								*P* value*
	*n*	%	*n*	%	*n*	%	*n*	%	
	< 0·023		0·023–0·045		0·046–0·095		> 0·096		
Number of participants	1299		1267		1283		1277		
Age (years), *n* (%)									< 0·001
35–44	195	15·0	113	8·9	125	9·7	130	10·2	
45–54	303	23·3	284	22·4	289	22·5	253	19·8	
55–64	519	40·0	562	44·4	551	43·0	560	43·9	
65–69	282	21·7	308	24·3	318	24·8	334	26·2	
Residential area, *n* (%)									< 0·001
Shiga	284	21·9	214	16·9	214	16·7	183	14·3	
Fukuoka	379	29·2	487	38·4	446	34·8	489	38·3	
Kyushu or Okinawa	636	49·0	566	44·7	623	48·6	605	47·4	
Educational attainment, *n* (%)^†^									0·08
Elementary or junior high school	201	15·5	140	11·1	145	11·3	143	11·2	
High school or junior college	42	3·2	27	2·1	32	2·5	19	1·5	
University or graduate school	37	2·9	43	3·4	35	2·7	20	1·6	
Others or missing	1019	78·4	1057	83·4	1071	83·5	1095	85·8	
Smoking exposure (pack-years), *n* (%)^‡^									< 0·001
0	445	34·3	415	32·8	341	26·6	298	23·3	
0·1–24·9	272	20·9	224	17·7	205	16·0	192	15·0	
25·0–41·1	197	15·2	236	18·6	229	17·9	237	18·6	
≥ 41·2	139	10·7	180	14·2	247	19·3	329	25·8	
Missing	246	18·9	212	16·7	261	20·3	221	17·3	
Alcohol consumption (g/d), *n* (%)^‡^									0·035
0·0	357	27·9	325	25·7	346	27·0	399	31·3	
0·1–19·5	329	25·3	312	24·6	297	23·2	270	21·1	
19·6–43·5	315	24·3	322	25·4	333	26·0	289	22·6	
≥ 43·6	298	22·9	308	24·3	307	23·9	319	25·0	
Total physical activity (MET-h/d), *n* (%)^§^									< 0·001
< 8·9	363	27·9	425	33·5	454	35·4	464	36·3	
9·0–27·4	432	33·3	423	33·4	424	33·1	425	33·3	
≥ 27·5	404	38·8	418	33·0	405	31·6	387	30·3	
Missing	0	0·0	1	0·1	0	0·0	1	0·1	
BMI (kg/m^2^)									
Median	22·6		23·6		24·2		24·5		< 0·001
IQR	20·8–24·3		22·1–25·5		22·4–26·2		22·6–26·6		
Total energy intake (kcal/d), *n* (%)^§^									0·08
< 1752	312	24·0	309	24·4	326	25·4	335	26·2	
1752–1941	329	25·3	301	23·8	310	24·2	341	26·7	
1942–2169	299	23·0	337	26·6	328	25·6	318	24·9	
≥ 2170	359	27·6	320	25·3	319	24·9	283	22·2	
	Median	IQR	Median	IQR	Median	IQR	Median	IQR	
Rice consumption (g/d), Median (IQR)^||^	472·4	358·1–601·0	458·1	358·1–601·0	458·1	358·1–601·0	443·9	358·1–601·0	0·033
Bread consumption (g/d), Median (IQR)^||^	18·1	1·0–81·0	18·1	1·0–81·0	18·1	1·0–81·0	18·1	1·0–81·0	0·14
Red meat consumption (g/d), Median (IQR)^||^	13·8	13·8–31·0	13·8	13·8–31·0	13·8	13·8–31·0	13·8	13·8–31·0	0·86
Processed meat consumption (g/d), Median (IQR)^||^	5·3	2·3–5·3	5·3	2·3–5·3	5·3	2·3–5·3	5·3	2·3–5·3	0·48
Poultry consumption (g/d), Median (IQR)^||^	12·8	12·8–28·5	12·8	4·7–12·8	12·8	4·7–12·8	12·8	4·7–12·8	0·07
Dairy products consumption (g/d), Median (IQR)^||^	56·6	11·7–161·0	56·6	11·7–161·0	56·6	11·7–161·0	56·6	11·7–161·0	0·86
Fish consumption (g/d), Median (IQR)^||^	39·0	19·0–51·4	39·0	20·3–56·0	39·0	20·3–57·7	39·0	20·3–58·5	0·14
Vegetables consumption (g/d), Median (IQR)^||^	108·4	70·0–155·5	107·9	71·4–155·6	107·7	68·4–153·8	110·3	70·9–158·6	0·71
Fruit consumption (g/d), Median (IQR)^||^	37·4	12·4–63·1	37·4	12·4–63·1	37·4	12·4–63·1	25·6	12·4–63·1	0·98
Nuts consumption (g/d), Median (IQR)^||^	2·3	1·0–5·3	2·3	1·0–5·3	2·3	1·0–5·3	2·3	1·0–2·3	0·001
Coffee consumption (ml/d), Median (IQR)^||^	151·0	22·4–251·0	151·0	22·4–251·0	251·0	22·4–251·0	251·0	22·4–251·0	0·13
Green tea consumption (ml/d), Median (IQR)^||^	441·0	111·0–1101·0	441·0	111·0–1101·0	441·0	111·0–1101·0	441·0	111·0–1101·0	0·11

Females									
Characteristics	Quartiles of hsCRP (mg/dl)								*P* value*
	*n*	%	*n*	%	*n*	%	*n*	%	
	< 0·015		0·015–0·031		0·032–0·070		> 0·070		
Number of participants	2163		2140		2109		2139		
Age (years), *n* (%)									< 0·001
35–44	544	25·2	273	12·8	201	9·5	195	9·2	
45–54	602	27·8	518	24·2	438	20·8	396	18·8	
55–64	763	35·2	969	45·3	1003	47·6	979	46·4	
65–69	254	11·7	380	17·8	466	22·1	541	25·6	
Residential area, *n* (%)									< 0·001
Shiga	520	24·0	469	21·9	409	19·4	356	16·9	
Fukuoka	509	23·5	709	33·1	701	33·3	646	30·6	
Kyushu or Okinawa	1134	52·4	962	45·0	998	47·3	1109	52·5	
Educational attainment, *n* (%)^†^									< 0·001
Elementary or junior high school	328	15·2	321	15·0	291	13·8	278	13·2	
High school or junior college	160	7·4	115	5·4	93	4·4	56	2·7	
University or graduate school	23	1·1	27	1·3	15	0·7	15	0·7	
Others or missing	1652	76·4	1677	78·4	1709	81·1	1762	83·5	
Smoking exposure (pack-years), *n* (%)^‡^									< 0·001
0	1837	84·9	1875	87·6	1865	88·5	1852	87·7	
0·1–24·9	94	4·4	72	3·4	51	2·4	50	2·4	
25·0–41·1	82	3·8	60	2·8	60	2·9	62	2·9	
≥ 41·2	58	2·7	61	2·9	69	3·3	83	3·9	
Missing	92	4·3	72	3·4	63	3·0	64	3·0	
Alcohol consumption (g/d), *n* (%)^‡^									< 0·001
0·0	1526	70·6	1560	72·9	1606	76·2	1602	75·9	
0·1–19·5	208	9·6	177	8·3	165	7·8	189	9·0	
19·6–43·5	217	10·0	202	9·4	167	7·9	147	7·0	
≥ 43·6	212	9·8	201	9·4	170·	8·1	173	8·2	
Total physical activity (MET-h/d), *n* (%)^§^									0·15
< 10·8	694	32·1	730	34·1	670·	31·8	740	35·1	
10·8–22·3	731	33·8	691	32·3	742	35·2	680	32·2	
≥ 22·4	738	34·1	718	33·6	694	32·9	688	32·6	
Missing	0	0·0	1	0·1	2	0·1	3	0·1	
BMI (kg/m^2^)									
Median	20·7		21·8		23·0		24·1		< 0·001
IQR	19·2–22·5		20·3–23·7		21·1–25·1		21·9–27·0		
Total energy intake (kcal/d), *n* (%)^§^									0·17
< 1752	540	25·0	549	25·7	516	24·5	525	24·9	
1752–1941	515	23·8	505	23·6	574	27·2	537	25·4	
1942–2169	536	24·8	538	25·1	513	24·3	544	25·8	
≥ 2170	572	26·4	548	25·6	505	24·0	505	23·9	
	Median	IQR	Median	IQR	Median	IQR	Median	IQR	
Rice consumption (g/d), Median (IQR)^||^	301·0	226·0–386·7	301·0	226·0–418·9	301·0	235·9–418·9	301·0	246·6–418·9	< 0·001
Bread consumption (g/d), Median (IQR)^||^	40·2	10·2–71·0	36·0	5·6–71·0	36·0	5·6–71·0	31·0	1·0–71·0	< 0·001
Red meat consumption (g/d), Median (IQR)^||^	12·8	12·8–28·5	12·8	12·8–28·5	12·8	12·8–28·5	12·8	12·8–28·5	0·036
Processed meat consumption (g/d), Median (IQR)^||^	5·3	2·3–5·3	5·3	2·3–5·3	5·3	2·3–5·3	5·3	2·3–5·3	< 0·001
Poultry consumption (g/d), Median (IQR)^||^	12·8	12·8–28·5	12·8	12·8–28·5	12·8	12·8–28·5	12·8	12·8–28·5	< 0·001
Dairy products consumption (g/d), Median (IQR)^||^	128·5	51·0–178·5	122·8	51·0–178·9	128·5	51·0–179·8	128·5	40·9–178·5	0·61
Fish consumption (g/d), Median (IQR)^||^	33·7	18·7–46·0	35·9	19·2–50·8	35·9	20·2–49·8	35·9	20·2–51·4	< 0·001
Vegetables consumption (g/d), Median (IQR)^||^	148·2	103·0–207·4	151·7	108·3–204·9	152·6	111·0–204·5	149·1	106·1–203·8	0·30
Fruit consumption (g/d), Median (IQR)^||^	37·4	18·1–86·0	60·3	24·2–100·3	60·3	25·6–108·1	60·3	25·6–100·3	0·001
Nuts consumption (g/d), Median (IQR)^||^	2·3	1·0–2·3	2·3	1·0–5·3	2·3	1·0–5·3	2·3	1·0–2·3	< 0·001
Coffee consumption (ml/d), Median (IQR)^||^	251·0	51·0–251·0	251·0	22·4–251·0	251·0	22·4–251·0	251·0	22·4–251·0	0·002
Green tea consumption (ml/d), Median (IQR)^||^	401·0	401·0–1001·0	401·0	401·0–1001·0	401·0	401·0–1001·0	401·0	401·0–1001·0	0·20

hsCRP, high-sensitivity C-reactive protein; MET-h/d, metabolic equivalent hours per d; IQR, interquartile range.

* Characteriscitcs of participants were compared between quartiles of hsCRP using the Kruskal–Wallis test for continuous variables and the *χ*^2^ of independence for categorical variables.

† Fukuoka and Kyushu or Okinawa area had no data about educational attainment.

‡ These covariates were divided into zero and tertiles above zero.

§ Total physical activity and total energy intake were divided into tertiles and quartiles, respectively.

|| Food group intakes were energy-unadjusted values, which had many tied values, especially among food groups consisting of few items.

Two research areas did not collect data on educational attainment, and thus, all the participants surveyed in those areas were categorised as missing for this covariate. Smoking exposure was calculated as pack-years as follows: (number of smoked cigarettes per d) × (duration of habitual smoking in years)/20. Alcohol consumption was calculated as g/d using the dose and frequency of drinking of each alcohol beverage. Total physical activity was calculated as MET-h/d using the intensity, duration and frequency of three levels of daily total or leisure-time physical activity (daily total physical activity consists of strenuous activity, walking and standing; leisure-time physical activity consists of vigorous, moderate and light activity). BMI was calculated as weight divided by height in metres squared (kg/m^2^). Self-reported weight and/or height were used to calculate BMI for 337 participants whose weight and/or height had not been measured. Five participants were excluded from the analysis because their self-reported height and/or weight were missing (Figure).

## Statistical analysis

All statistical analyses were stratified by sex. Participant characteristics were expressed as geometric means (95% CI) for hsCRP or medians (interquartile ranges) for other continuous variables, and as numbers and proportions (%) for categorical variables. Associations between participant characteristics and the quartiles of log-transformed hsCRP concentrations were examined using the Kruskal–Wallis test for continuous variables because of deviation from normality on quantile–quantile plots or unequal variances. The *χ* ^2^ test of independence was adopted for categorical variables. Additionally, characteristics were compared between participants included in the present analysis and those excluded because their total energy intake deviated from the mean of their sex or because they had missing data for at least one item necessary to calculate food group intakes. These comparisons used the Mann–Whitney test for continuous variables and the *χ* ^2^ test of independence for categorical variables.

Food group intakes were log-transformed and energy-adjusted using the residual method^(20)^, and Pearson’s correlation coefficients were calculated between food group intakes. The present study defined Pearson’s correlation coefficients of 0·00–0·09 as negligible, 0·10–0·39 as weak, 0·40–0·69 as moderate, 0·70–0·89 as strong and 0·90–1·00 as very strong^(21)^. Energy-adjusted food group intakes were then categorised into quartiles. Their cut-off values were calculated by exponentiating the estimates of the residual method, which were the sum of an expected log-transformed food group intake for the mean total energy intake and a residual of a log-transformed food group intake^(20)^.

Associations between the quartiles of energy-adjusted food group intakes and log-transformed hsCRP were examined using multiple linear regression, and their exponentiated coefficients for log-transformed hsCRP were estimated with their 95 % CI. Food group intakes were categorised into quartiles to deal with possible non-linear associations with hsCRP, and quartile 1 was treated as a reference category. Exponentiated coefficients indicate the concentration ratios of hsCRP comparing quartiles 2–4 with quartile 1 of food group intake. The present study established the following three models: (1) a model that incorporated one food group as an exposure and was adjusted for age and total energy intake, (2) a model that was further adjusted for all the covariates listed in the section ‘covariates’ and (3) a model that further incorporated all the other food groups as exposures. All the multivariable models incorporated missing categories for the covariates with missing values (educational attainment, smoking exposure, alcohol consumption and total physical activity). According to a recommendation of a methodological review, both models 2 and 3 were established because model 3 eliminated potential confounding from food groups other than an exposure, but the result may not have been applicable to the whole population because of restrictions in underlying food patterns^(22)^. To address a concern about model 3’s excessive adjustment, we calculated variance inflation factors in model 3. Furthermore, an additional analysis was conducted among food groups whose *P* trends turned from statistically significant to non-significant after adjusting for other food groups. This additional analysis incorporated only one food group as a covariate based on their correlation (Pearson’s correlation coefficients of 0·10 or higher in absolute values) with exposures of interest. *P* trend values were also calculated by entering ordinal variables defined as 0, 1, 2 and 3 for quartiles 1–4 of food group intakes, respectively, as continuous variables in the models.

As a sensitivity analysis, multiple imputation was conducted in each sex by combining 868 males and 1470 females excluded from the main analysis using the complete data with 5126 males and 8522 females included in the main analysis. The former participants had missing data for at least one item necessary to calculate food group intakes but satisfied no other exclusion criteria. See Supplemental Text for the detailed methods of multiple imputation. All statistical tests were two-sided, and *P* < 0·05 was considered statistically significant. Stata 13.1 and 17.0 (StataCorp) was used for statistical analyses.

## Results

Table 1 shows the participants’ characteristics according to the quartiles of hsCRP by sex. In males, those with a higher hsCRP concentration tended to be older, to live in Fukuoka, to have higher smoking exposure, to be physically inactive and to have a higher BMI. Regarding food groups, males with a higher hsCRP concentration were more likely to have a lower intake of rice and nuts. In females, those with a higher hsCRP concentration tended to be older, to live in Fukuoka, to have lower educational attainment and higher smoking exposure, to drink less alcohol and to have a higher BMI. Regarding food groups, females with a higher hsCRP concentration were more likely to have higher intakes of rice, fish and fruit and lower intakes of bread, red and processed meat, poultry, nuts and coffee. The exclusion of potential participants according to deviations in total energy intake was not strongly associated with the values of study variables (see online supplementary material, Supplemental Table 1 and Section A of Supplemental Text). However, associations were clear between exclusions according to missingness in at least one item necessary to calculate food group intakes and the values of study variables (see online supplementary material, Supplemental Table 2 and Section A of Supplemental Text).

Pearson’s correlation coefficients showed moderate-strength correlations between red meat and poultry intake in both sexes and between vegetables and fruit in males (see online supplementary material, Supplemental Table 3). Online Supplemental Table 4 shows the estimated ranges of energy-adjusted intakes of food groups by sex. The geometric mean of hsCRP was 0·048 mg/dl (95 % CI 0·047, 0·050) in males and 0·034 mg/dl (95 % CI 0·034, 0·035) in females.

Table 2 shows associations between the quartiles of food group intakes and hsCRP concentrations by sex. In males, model 3, which incorporated all food groups as exposures, provided significant concentration ratios of hsCRP comparing quartiles 4 and 1 among three food group intakes: 1·12 (95 % CI 1·02, 1·22; *P* trend = 0·014) for processed meat, 1·13 (95 % CI 1·03, 1·24; *P* trend = 0·025) for fish and 0·83 (95 % CI 0·76, 0·90; *P* trend < 0·001) for nuts. The results of model 3 were almost the same as those of model 2, which incorporated only one food group intake as an exposure. In females, model 3 showed two positive concentration ratios: 1·11 (95 % CI 1·03, 1·19; *P* trend = 0·002) for processed meat and 1·19 (95 % CI 1·11, 1·29; *P* trend < 0·001) for fruit. On the other hand, inverse correlations were found in following four concentration ratios: 0·89 (95 % CI 0·81, 0·97; *P* trend = 0·010) for bread, 0·86 (95 % CI 0·80, 0·92; *P* trend < 0·001) for vegetables, 0·90 (95 % CI 0·84, 0·97; *P* trend = 0·001) for nuts and 0·88 (95 % CI 0·82, 0·95; *P* trend < 0·001) for green tea. The *P* trend for rice and poultry was significant in model 2, but non-significant in model 3, and vice versa for processed meat. Variance inflation factor did not indicate excessive multicollinearity in both sexes; they were smaller than four for all the ordinal and continuous variables (see online supplementary material, Supplemental Table 5). In an additional analysis, which incorporated only one food group as a covariate, model 2’s statistically significant *P* trend was diluted in rice but retained in poultry (see online supplementary material, Supplemental Table 6). The directions of the associations were consistent between the analysis using complete data and that using multiply imputed data (Table 2, see online supplementary material, Supplemental Table 7 and Section C of Supplemental Text).

**Table 2:** Associations between energy-adjusted intakes (residuals) of each food groups and hsCRP by sex

Males												
Food group	Residual quartile of log-transformed food group consumption	hsCRP (mg/dl), geometric mean	95 % CI	Model 1*		*P* trend	Model 2^†^		*P* trend	Model 3^‡^		*P* trend
				Exponentiated coefficient^§^	95 % CI		Exponentiated coefficient^§^	95 % CI		Exponentiated coefficient^§^	95 % CI	
Rice	Quartile 1	0·048	0·045, 0·051	Reference		0·51	Reference		0·82	Reference		0·70
	Quartile 2	0·049	0·046, 0·052	1·03	0·95, 1·12		1·05	0·97, 1·15		1·04	0·96, 1·14	
	Quartile 3	0·048	0·045, 0·051	0·99	0·91, 1·07		1·02	0·93, 1·11		1·00	0·91, 1·10	
	Quartile 4	0·049	0·046, 0·052	0·98	0·90, 1·07		1·02	0·93, 1·12		0·99	0·87, 1·12	
Bread	Quartile 1	0·052	0·049, 0·055	Reference		0·11	Reference		0·09	Reference		0·11
	Quartile 2	0·046	0·043, 0·049	0·93	0·85, 1·02		0·91	0·83, 0·99		0·90	0·82, 0·99	
	Quartile 3	0·049	0·046, 0·052	0·97	0·89, 1·06		0·96	0·88, 1·04		0·95	0·86, 1·04	
	Quartile 4	0·046	0·044, 0·049	0·91	0·84, 1·00		0·90	0·83, 0·99		0·90	0·80, 1·00	
Red meat	Quartile 1	0·047	0·044, 0·049	Reference		0·09	Reference		0·10	Reference		0·15
	Quartile 2	0·048	0·045, 0·051	1·06	0·97, 1·16		1·07	0·98, 1·17		1·09	0·99, 1·20	
	Quartile 3	0·049	0·046, 0·052	1·03	0·95, 1·13		1·02	0·94, 1·12		1·07	0·97, 1·18	
	Quartile 4	0·050	0·047, 0·053	1·09	1·00, 1·19		1·09	1·00, 1·19		1·10	0·99, 1·21	
Processed meat	Quartile 1	0·048	0·045, 0·051	Reference		0·018	Reference		0·021	Reference		0·014
	Quartile 2	0·047	0·045, 0·050	0·97	0·89, 1·06		0·97	0·89, 1·06		0·99	0·90, 1·08	
	Quartile 3	0·047	0·044, 0·049	0·98	0·90, 1·07		0·99	0·91, 1·08		1·01	0·92, 1·10	
	Quartile 4	0·052	0·049, 0·055	1·11	1·02, 1·21		1·10	1·01, 1·20		1·12	1·02, 1·22	
Poultry	Quartile 1	0·051	0·048, 0·054	Reference		0·30	Reference		0·70	Reference		0·26
	Quartile 2	0·047	0·044, 0·050	1·00	0·91, 1·10		1·01	0·92, 1·11		0·99	0·90, 1·10	
	Quartile 3	0·048	0·045, 0·051	0·91	0·83, 1·00		0·93	0·85, 1·02		0·91	0·82, 1·01	
	Quartile 4	0·047	0·044, 0·050	0·98	0·90, 1·06		1·00	0·92, 1·09		0·96	0·87, 1·06	
Dairy products	Quartile 1	0·047	0·044, 0·050	Reference		0·69	Reference		0·41	Reference		0·29
	Quartile 2	0·049	0·046, 0·052	1·03	0·95, 1·12		1·04	0·95, 1·13		1·05	0·96, 1·14	
	Quartile 3	0·049	0·047, 0·053	1·04	0·96, 1·13		1·08	0·99, 1·17		1·08	1·00, 1·18	
	Quartile 4	0·048	0·045, 0·051	0·98	0·90, 1·06		1·03	0·94, 1·12		1·04	0·95, 1·14	
Fish	Quartile 1	0·045	0·043, 0·048	Reference		0·039	Reference		0·013	Reference		0·025
	Quartile 2	0·048	0·046, 0·051	1·06	0·98, 1·15		1·06	0·98, 1·15		1·06	0·97, 1·15	
	Quartile 3	0·047	0·044, 0·050	0·99	0·91, 1·08		1·00	0·92, 1·09		1·01	0·92, 1·10	
	Quartile 4	0·053	0·050, 0·056	1·13	1·03, 1·23		1·14	1·05, 1·25		1·13	1·03, 1·24	
Vegetables	Quartile 1	0·049	0·046, 0·052	Reference		0·70	Reference		0·60	Reference		0·85
	Quartile 2	0·047	0·044, 0·050	0·95	0·87, 1·03		0·96	0·89, 1·05		0·95	0·87, 1·04	
	Quartile 3	0·048	0·045, 0·050	0·96	0·88, 1·04		0·98	0·90, 1·07		0·96	0·88, 1·05	
	Quartile 4	0·050	0·047, 0·053	0·98	0·90, 1·07		1·02	0·94, 1·11		0·98	0·89, 1·08	
Fruit	Quartile 1	0·047	0·044, 0·050	Reference		0·29	Reference		0·96	Reference		0·90
	Quartile 2	0·050	0·047, 0·053	1·01	0·92, 1·10		1·05	0·96, 1·14		1·07	0·98, 1·16	
	Quartile 3	0·048	0·045, 0·051	0·98	0·90, 1·06		1·02	0·93, 1·11		1·03	0·95, 1·13	
	Quartile 4	0·048	0·046, 0·051	0·96	0·88, 1·05		1·01	0·92, 1·10		1·02	0·92, 1·12	
Nuts	Quartile 1	0·052	0·049, 0·055	Reference		< 0·001	Reference		< 0·001	Reference		< 0·001
	Quartile 2	0·050	0·047, 0·053	0·95	0·86, 1·03		0·94	0·86, 1·03		0·95	0·87, 1·04	
	Quartile 3	0·047	0·044, 0·050	0·84	0·77, 0·92		0·85	0·78, 0·93		0·85	0·78, 0·94	
	Quartile 4	0·045	0·042, 0·047	0·82	0·75, 0·90		0·83	0·77, 0·91		0·83	0·76, 0·90	
Coffee	Quartile 1	0·048	0·045, 0·050	Reference		0·11	Reference		0·38	Reference		0·44
	Quartile 2	0·047	0·044, 0·050	1·01	0·93, 1·10		1·01	0·93, 1·10		1·02	0·93, 1·11	
	Quartile 3	0·051	0·048, 0·054	1·07	0·98, 1·16		1·00	0·92, 1·09		1·01	0·92, 1·10	
	Quartile 4	0·048	0·045, 0·050	1·06	0·97, 1·15		0·96	0·88, 1·05		0·96	0·88, 1·06	
Green tea	Quartile 1	0·050	0·047, 0·053	Reference		0·025	Reference		0·07	Reference		0·048
	Quartile 2	0·048	0·045, 0·051	0·96	0·88, 1·05		0·97	0·89, 1·06		0·97	0·89, 1·06	
	Quartile 3	0·047	0·045, 0·050	0·92	0·84, 1·00		0·92	0·84, 1·00		0·92	0·85, 1·01	
	Quartile 4	0·048	0·045, 0·051	0·92	0·84, 1·00		0·94	0·86, 1·02		0·93	0·85, 1·01	

Females												
Food group	Residual quartile of log-transformed food group consumption	hsCRP (mg/dl), geometric mean	95 % CI	Model 1*		*P* trend	Model 2^†^		*P* trend	Model 3^‡^		*P* trend
				Exponentiated coefficient^§^	95 % CI		Exponentiated coefficient^§^	95 % CI		Exponentiated coefficient^§^	95 % CI	
Rice	Quartile 1	0·031	0·030, 0·033	Reference		< 0·001	Reference		< 0·001	Reference		0·07
	Quartile 2	0·033	0·032, 0·035	1·05	0·99, 1·13		1·07	1·00, 1·14		1·06	0·99, 1·14	
	Quartile 3	0·035	0·033, 0·036	1·06	0·99, 1·14		1·06	0·99, 1·14		1·06	0·97, 1·14	
	Quartile 4	0·039	0·037, 0·041	1·16	1·08, 1·24		1·17	1·09, 1·26		1·13	1·02, 1·25	
Bread	Quartile 1	0·039	0·038, 0·041	Reference		< 0·001	Reference		< 0·001	Reference		0·010
	Quartile 2	0·034	0·032, 0·036	0·93	0·87, 1·00		0·93	0·86, 0·99		0·95	0·89, 1·02	
	Quartile 3	0·033	0·032, 0·035	0·93	0·87, 0·99		0·93	0·86, 0·99		0·96	0·89, 1·04	
	Quartile 4	0·031	0·030, 0·033	0·86	0·80, 0·92		0·85	0·79, 0·91		0·89	0·81, 0·97	
Red meat	Quartile 1	0·036	0·035, 0·038	Reference		0·31	Reference		0·30	Reference		0·96
	Quartile 2	0·035	0·034, 0·037	0·96	0·89, 1·03		0·96	0·89, 1·03		0·97	0·90, 1·05	
	Quartile 3	0·032	0·030, 0·033	0·93	0·87, 0·99		0·93	0·87, 1·00		0·96	0·89, 1·03	
	Quartile 4	0·034	0·033, 0·036	0·97	0·91, 1·04		0·97	0·91, 1·04		1·00	0·93, 1·08	
Processed meat	Quartile 1	0·036	0·035, 0·038	Reference		0·07	Reference		0·07	Reference		0·002
	Quartile 2	0·034	0·032, 0·036	0·99	0·92, 1·06		0·99	0·92, 1·06		1·00	0·94, 1·08	
	Quartile 3	0·034	0·033, 0·036	1·02	0·95, 1·09		1·02	0·95, 1·09		1·05	0·98, 1·13	
	Quartile 4	0·033	0·032, 0·035	1·06	0·99, 1·13		1·06	0·99, 1·13		1·11	1·03, 1·19	
Poultry	Quartile 1	0·037	0·035, 0·039	Reference		0·007	Reference		0·016	Reference		0·07
	Quartile 2	0·035	0·033, 0·037	0·97	0·91, 1·04		0·98	0·91, 1·05		0·97	0·91, 1·05	
	Quartile 3	0·033	0·032, 0·035	0·93	0·87, 1·00		0·94	0·88, 1·01		0·97	0·90, 1·04	
	Quartile 4	0·032	0·031, 0·034	0·92	0·86, 0·98		0·93	0·87, 0·99		0·94	0·88, 1·02	
Dairy products	Quartile 1	0·036	0·034, 0·038	Reference		0·28	Reference		0·25	Reference		0·72
	Quartile 2	0·033	0·031, 0·034	0·91	0·85, 0·97		0·91	0·85, 0·97		0·92	0·86, 0·98	
	Quartile 3	0·034	0·033, 0·036	0·95	0·89, 1·01		0·95	0·89, 1·02		0·97	0·90, 1·03	
	Quartile 4	0·035	0·033, 0·036	0·95	0·89, 1·01		0·95	0·89, 1·01		0·97	0·91, 1·05	
Fish	Quartile 1	0·032	0·030, 0·033	Reference		0·58	Reference		0·43	Reference		0·22
	Quartile 2	0·033	0·032, 0·035	1·00	0·94, 1·07		1·01	0·94, 1·08		1·01	0·94, 1·08	
	Quartile 3	0·034	0·033, 0·036	0·96	0·90, 1·03		0·97	0·91, 1·04		0·98	0·92, 1·05	
	Quartile 4	0·039	0·037, 0·040	1·03	0·97, 1·11		1·04	0·97, 1·12		1·05	0·98, 1·13	
Vegetables	Quartile 1	0·034	0·032, 0·036	Reference		0·003	Reference		0·002	Reference		< 0·001
	Quartile 2	0·035	0·033, 0·036	0·99	0·92, 1·05		0·98	0·92, 1·05		0·97	0·91, 1·04	
	Quartile 3	0·036	0·034, 0·038	0·99	0·93, 1·06		0·99	0·93, 1·06		0·96	0·90, 1·03	
	Quartile 4	0·033	0·032, 0·035	0·90	0·84, 0·96		0·89	0·84, 0·96		0·86	0·80, 0·92	
Fruit	Quartile 1	0·030	0·029, 0·032	Reference		0·012	Reference		0·005	Reference		< 0·001
	Quartile 2	0·034	0·032, 0·035	1·05	0·98, 1·12		1·05	0·98, 1·12		1·08	1·01, 1·16	
	Quartile 3	0·036	0·035, 0·038	1·09	1·02, 1·17		1·10	1·03, 1·18		1·17	1·09, 1·26	
	Quartile 4	0·038	0·036, 0·040	1·08	1·01, 1·16		1·10	1·02, 1·17		1·19	1·11, 1·29	
Nuts	Quartile 1	0·036	0·035, 0·038	Reference		< 0·001	Reference		0·001	Reference		0·001
	Quartile 2	0·034	0·032, 0·036	0·96	0·89, 1·03		0·96	0·89, 1·03		0·95	0·89, 1·03	
	Quartile 3	0·033	0·031, 0·034	0·88	0·82, 0·94		0·88	0·82, 0·94		0·88	0·82, 0·94	
	Quartile 4	0·034	0·033, 0·036	0·90	0·84, 0·96		0·90	0·84, 0·97		0·90	0·84, 0·97	
Coffee	Quartile 1	0·038	0·036, 0·039	Reference		0·39	Reference		0·13	Reference		0·35
	Quartile 2	0·033	0·031, 0·034	0·91	0·85, 0·97		0·94	0·87, 1·01		0·95	0·89, 1·02	
	Quartile 3	0·036	0·034, 0·037	1·01	0·94, 1·08		0·99	0·93, 1·06		1·01	0·94, 1·09	
	Quartile 4	0·032	0·030, 0·033	0·93	0·87, 1·00		0·93	0·86, 0·99		0·94	0·88, 1·01	
Green tea	Quartile 1	0·033	0·031, 0·035	Reference		< 0·001	Reference		0·001	Reference		< 0·001
	Quartile 2	0·035	0·033, 0·037	0·95	0·89, 1·02		0·97	0·91, 1·04		0·96	0·90, 1·03	
	Quartile 3	0·034	0·033, 0·036	0·91	0·85, 0·98		0·93	0·87, 1·00		0·92	0·86, 0·99	
	Quartile 4	0·035	0·034, 0·037	0·88	0·82, 0·95		0·90	0·84, 0·96		0·88	0·82, 0·95	

hsCRP, high-sensitivity C-reactive protein; MET-h/d, metabolic equivalent hours per d.

* Adjusted for age (35–44, 45–54, 55–64, 65–69 years) and total energy intake (males: < 1752, 1752–1941, 1942–2169, ≥ 2170 kcal/d; females:< 1443, 1443–1574, 1575–1701, ≥ 1702 kcal/d).

† Adjusted for confounding factors in model 1 and residential area (Shiga, Fukuoka, Kyushu or Okinawa), educational attainment (elementary or junior high school, high school or junior college, university or graduate school, others or missing), smoking exposure (males: 0, 0·1–24·9, 25·0–41·1, ≥ 41·2 pack-years, missing; females: 0, 0·1–9·1, 9·2–19·9, ≥ 20·0 pack-years, missing), alcohol consumption (males: 0·0, 0·1–19·5, 19·6–43·5, ≥ 43·6 g/d; females: 0·0, 0·1–4·7, 4·8–13·7, ≥ 13·8 g/d) and total physical activity (males: < 8·9, 9·0–27·4, ≥ 27·5 MET-h/d, missing; females: < 10·8, 10·8–22·3, ≥ 22·4 MET-h/d, missing).

‡ Adjusted for confounding factors in model 2 and the residual quartiles of other log-transformed food group consumption.

§ Exponentiated coefficients are ratios of estimated hsCRP concentration for the food group consumption of quartiles 2–4 to that for quartile 1.

## Discussion

Previous dietary pattern analyses have demonstrated that healthy dietary patterns can lower hsCRP concentrations^(3,4)^. These patterns have common characteristics of higher intakes of fruit, vegetables, fish, nuts and whole grain combined with lower intakes of red meat, processed foods and refined grain. The present study confirmed the associations among processed meat, vegetables and nuts reported by dietary pattern analyses. Interestingly, however, the present study found that bread intake was inversely and fish and fruit intakes positively associated with hsCRP concentrations.

The inconsistent findings of the present study compared with those of the previous dietary pattern analyses may have derived from differences in dietary patterns between the former and the latter. The present study found weak positive correlations among fruit, vegetables and fish but also found positive or no correlations between these food groups and red and processed meat, which differs from those suggested by previous dietary pattern analyses (see online supplementary material, Supplemental Table 3). Moreover, the present participants had high intakes of rice and green tea (see online supplementary material, Supplemental Table 4), which are also distinct from the dietary patterns reported in a previous study^(10)^. Other possible explanations for the inconsistency with previous dietary pattern analyses are the differences in genetic backgrounds and the accompanying responsiveness of inflammatory processes between the study participants.

In the present study, rice and bread intake would have been mostly occupied by white rice and bread because the consumption of whole grain has been reported to be low among East Asian populations, including Japanese^(23)^. Although whole grain intake has been linked to reduced inflammation levels in a meta-analysis of RCT^(24)^, few studies have examined the effects of refined grain on chronic inflammation. However, both white rice and white bread have a high glycaemic index^(25)^, which has been associated with higher inflammation levels and chronic diseases in systematic reviews of observational studies^(26)^. The present analysis showed that rice marginally raised, but bread lowered, hsCRP concentrations in females. Three factors could explain this inconsistency. First, the estimated bread intake was far lower than rice intake in the present study (see online supplementary material, Supplemental Table 4), and thus, bread intake may have been insufficient to exert its effect of a high glycemic index. Second, dietary fibre has been shown to improve the degree of inflammation in experimental and interventional studies^(27)^. The dose of dietary fibre in white bread (4·2 g/100 g) is higher than that in rice (1·5 g/100 g)^(28)^. Thus, in white bread, the protective effect of dietary fibre on inflammation may surpass the negative effect of its high glycaemic index, and even a relatively low intake of white bread might be sufficient to suppress inflammation.

Red and processed meat has been shown to promote inflammation in several observational studies^(29,30)^, but others have found an inverse or no association between poultry intake and chronic inflammation^(6,30)^. In the present study, processed meat increased hsCRP concentrations, whereas red meat and poultry had marginally positive and negative effects, respectively. Experimental studies have reported that exposing red meat to high temperatures or elevated pH levels produces advanced glycation end products, polycyclic aromatic hydrocarbons and heterocyclic amines, all of which can enhance inflammation^(31,32)^. Therefore, processed meat could have stronger potential to raise hsCRP concentrations compared with red meat and poultry. Additionally, poultry includes a lower content of heme iron and N-glycolylneuraminic acid than do beef and pork, and these substances can trigger inflammatory processes^(33)^. The non-significant effect of red meat on hsCRP concentrations in the present study may also have derived from lower red meat intakes (the cut-offs of quartile 4 were 27·3 and 27·2 g/d in males and females, respectively) compared with previous studies (the cut-offs of the highest category were 104 g/d in Ley et al.’s study^(29)^ and 97·7 g/d in van Woudenbergh et al.’s study^(30)^). Although the FFQ used in the present study tended to underestimate meat intake in individuals with higher consumption^(18)^, the differences in red meat intake between the present and previous studies seemed large (see online supplementary material, Supplemental Table 4).

Dairy products have been shown to have a neutral or a beneficial effect on inflammatory biomarkers in two meta-analyses of RCT^(34,35)^. The present study found no significant associations between dairy products intake and hsCRP, which partly agreed with the findings of previous studies. Although dairy products are rich in SFA, a systematic review of RCT showed that the fat content of dairy products may not affect inflammatory markers^(36)^.

Fish consumption has been linked to lower inflammatory biomarker concentrations in several observational studies^(37,38)^ and to reduced risks of coronary artery disease, stroke and all-cause mortality in two meta-analyses of prospective studies^(39,40)^. However, our analyses found that fish intake was correlated with higher hsCRP concentrations in males. The following factors could explain this discrepancy. First, the processing, shipping and storage of fish often involve high or low temperatures, light and oxygen, which degrade cholesterol to form cholesterol oxidation products and promote inflammation^(41)^. Second, fish contains methylmercury, a proinflammatory substance. Yet, a cross-sectional study reported that blood mercury levels were not associated with CRP concentrations after adjusting for fish intake^(38)^.

Fruit and vegetables have been shown to ameliorate inflammation in a meta-analysis of observational and interventional studies^(42)^. In the present study, however, fruit intake was related to a higher hsCRP concentration in females. Although fruit and vegetables contain many anti-inflammatory compounds such as dietary fibre, folate, vitamins and phytochemicals^(42)^, fruit also has high fructose content. Experimental studies have demonstrated that excessive fructose intake can enhance macrophage infiltration into adipocytes and promote the release of proinflammatory cytokines^(43)^. Additionally, excessive fructose consumption may induce weight gain^(43)^. In the present study, however, Spearman’s rank correlation coefficients showed negligible correlations between the residuals of log-transformed and energy-adjusted fruit intake and BMI (males: *r* = 0·000, *P* = 0·48; females: *r* = 0·04, *P* < 0·001). Further, a systematic review of meta-analyses revealed that raw or dried fruit can decrease the risk of chronic disease and predisposing factors, but that canned fruit may increase the risk of all-cause mortality^(44)^. Finally, the positive association between fruit intake and hsCRP may have derived from limitations of the present FFQ, which included only two items about fruit and did not distinguish raw fruit from canned fruit. These limitations may have led to a lower intake and negated the anti-inflammatory effect of raw fruit. The findings of the present study should be corroborated by cohort studies or RCT, and closer attention may need to be paid to the potential proinflammatory effects of fruit to recommend prudent fruit intake.

Few observational studies have reported the anti-inflammatory effects of nuts^(5,45)^, and these effects have not been confirmed by a meta-analysis of RCT^(46)^. However, nuts are rich in anti-inflammatory substances, including dietary fibre, Mg, phenolic compounds, unsaturated fatty acids and L-arginine^(45)^. A meta-analysis of observational studies has also reported that nuts can lower the risk of coronary artery disease^(39)^. Further studies are needed to confirm the potential anti-inflammatory effect of nuts.

In the present analyses, coffee intake had no effect on hsCRP concentrations in either sex, but green tea had an inverse relationship with hsCRP in females. Coffee contains anti-inflammatory and antioxidative compounds such as polyphenols, diterpenes, chlorogenic acids and caffeine^(47)^. Two meta-analyses of cross-sectional studies have investigated the association between coffee intake and CRP concentrations^(19,47)^, but only one found an inverse association^(19)^. The lack of an association found in a previous meta-analysis and the present analysis may have derived from several factors. First, coffee is often consumed with sugar, which could inhibit the anti-inflammatory and antioxidative potential of coffee. Second, individuals who consume higher amounts of coffee may have unhealthy lifestyles. However, in the present study, coffee intake was not associated with hsCRP concentrations, even after adjusting for lifestyle-related factors, including smoking and drinking (models 2 and 3). Third, the moderate coffee consumption seen in the present study may have led to a null association. Yet, a meta-analysis that found no association between coffee consumption and CRP levels included studies with the highest mean coffee intakes of 1037 and 1305 ml/d^(47)^. Green tea is a rich source of catechins, which also have anti-inflammatory and antioxidative effects. However, two meta-analyses of RCT reported finding no significant effects of green tea or green tea extracts, including catechins, on CRP concentrations^(48,49)^. The sample sizes of those two meta-analyses were 887 and 377, which may have led to lower statistical powers than that of the present study. On the other hand, the present results may have been affected by residual confounding from unmeasured confounding factors.

Model 2, which incorporated only one food group as an exposure, and model 3, which incorporated all the food groups as exposures, generally showed consistent results. In females, however, the *P* trends of model 3 were non-significant for rice and poultry, which differed from those of model 2 (Table 2). These diluted associations may have derived from an adjustment from correlated food groups, and this speculation was partly confirmed by an additional analysis adjusted for only one food group correlated with the examined food group, especially in rice (see online supplementary material, Supplemental Table 6). Whereas model 3 has the strength of eliminating potential confounding from other food groups, its limitation of fixing dietary patterns should be mentioned.

The present study has three main strengths: a relatively large sample of community-dwelling populations, the inclusion of multiple food groups as exposures and adjustments for potential confounding factors. Still, six limitations should be mentioned. First, reverse causation may have affected the present study. Although the present study excluded persons with hsCRP concentrations higher than 1·5 mg/dl and/or a history of coronary artery disease, stroke or cancer, we cannot infer causality only from the present results. Second, food group intakes were estimated using an FFQ, and thus, estimated intakes may have included some misclassification, especially in food groups with relatively low validity, such as meat and vegetables. However, food group intakes of the present study other than fruit were generally comparable to those of a study conducted in multiple areas of Japan^(50)^. Third, the data used in the present study included missing values for many variables. Yet, the results of multiple imputation analysis were materially unchanged from those of the main analysis (Table 2 and see online supplementary material, Supplemental Table 7). Fourth, residual confounding from unmeasured confounding factors may have produced the differences in the results between the previous RCT and our study. Fifth, the present study conducted multiple comparisons. However, since the present study did not aim to generalise the results of one food group to other food groups or those of one sex to another (individual testing), corrections for multiple testing are not appropriate^(51)^. Sixth, the present analyses were conducted using data from volunteering or participants in health checkups (the response proportion among eligible individuals was 38·5 %), who would be more likely to have high health-consciousness. Therefore, the correlations in general populations may differ from the findings of the present study.

The results of the present study have two implications for clinical practice and research. First, food group analyses can have the strength in clarifying which food groups composing currently proposed dietary patterns are influential on chronic inflammation. Second, our findings suggest that recommendations for individual food groups based on the results of dietary pattern analyses might require some modifications, especially for the Japanese population.

In conclusion, female bread intake was associated with lower and female fruit and male fish consumption was with higher hsCRP levels. These findings, combined with the results from dietary pattern analyses, would be informative to prevent chronic inflammation and its consequences.

## Supporting information

Matsunaga et al. supplementary material 1 Matsunaga et al. supplementary material

Matsunaga et al. supplementary material 2 Matsunaga et al. supplementary material

## Supplementary material

For supplementary material accompanying this paper visit https://doi.org/10.1017/S1368980024001599
