# Supplementary material for: Food group intakes and high-sensitivity C-reactive protein among community-dwelling Japanese adults: a cross-sectional study
Source: Public Health Nutr. 2024 Oct 18;27(1):e212. doi: 10.1017/S1368980024001599 (PMC11604322; doi:10.1017/S1368980024001599)
Supplement: Matsunaga et al. supplementary material 2 — Matsunaga et al. supplementary material [file S1368980024001599sup002.docx]

**Supplemental Text**

**Section A: Characteristics of participants excluded from the main analysis using complete data**

Compared with the participants included in the main analysis, those excluded because of their deviated total energy intake tended to live in Kyushu or Okinawa, drink more alcohol (males only), be physically active, have a higher energy intake, and have a lower hsCRP concentration (males only). These participants were also likely to have higher rice, processed meat (females only), fish (males only), vegetables (females only) and green tea (males only) intakes (Supplemental Table 1). Additionally, participants excluded because of their missingness for at least one item necessary to calculate food group intakes showed different characteristics from those included in the main analysis. The former tended to be younger, to live in Kyushu or Okinawa, to have lower or higher smoking exposure (males and females, respectively), to drink less alcohol, to be physically active, to have a higher BMI and a lower energy intake, and to have a higher hsCRP concentration (females only). The former was also likely to have a higher rice intake and lower bread, red meat, poultry, fruit, nuts, coffee, and green tea intakes (red meat, poultry, and fruit intakes decreased only in excluded females, and coffee intake decreased only in excluded males) (Supplemental Table 2).

**Section B: Methods of multiple imputation**

Multiple imputation was conducted because the values of food group intakes, hsCRP, and covariates were associated with exclusion from the main analysis using the complete data (Supplemental Table 2), which could have induced selection bias ^(1)^. The numbers of participants with missing data were as follows in males and females, respectively: 312 and 525 (rice), 382 and 651 (bread), 25 and 45 (red meat), 20 and 21 (processed meat), 51 and 53 (poultry), 141 and 214 (dairy), 71 and 81 (fish), 208 and 334 (vegetables), 22 and 35 (fruit), 59 and 75 (nuts), 21 and 27 (coffee), 16 and 15 (green tea), 5,025 and 8,106 (educational attainment), 1,228 and 392 (smoking exposure), 6 and 14 (total physical activity), and 3 and 7 (BMI) among 5,994 males and 9,992 females. All food group intake quartiles and three covariates (educational attainment, smoking exposure, and total physical activity) were imputed as ordinal variables using log-transformed hsCRP concentrations and other covariates (age, residential area, alcohol consumption, and total energy intake) in addition to BMI as an auxiliary variable. Multiple imputation by chained equations (MICE) was used with a burn-in period of 20, an imputation number of 100, and an augmentation method to deal with perfect prediction of ordinal variables. All food group intake quartiles and BMI values were imputed using ordinal logistic regression and linear regression, respectively. The covariates with missing data were imputed using ordinal logistic regression in males and predictive mean matching with a nearest neighbor number of five in females because an imputation using ordinal logistic regression was not completed in females. Imputed values converged, and the distributions of imputed values were consistent with those of observed values. Multiple imputation was conducted using the commands mi impute chained and mi estimate in Stata 17.0. The distributions of imputed values were compared with those of observed values using the command midiagplots ^(2)^.

1. Hughes RA, Heron J, Sterne JAC et al. (2019) Accounting for missing data in statistical analyses: multiple imputation is not always the answer. *Int J Epidemiol* **48**, 1294–1304.
2. Eddings W, Marchenko Y (2012) Diagnostics for multiple imputation in Stata. <https://ideas.repec.org/a/tsj/stataj/v12y2012i3p353-367.html> (Accessed Dec 2022).

**Section C: Results of multiple imputation analysis**

Multiple imputation analysis provided different results from the main analysis using complete data in the following food group intakes. In males, red meat intake showed a positive dose-response relationship with hsCRP, and the significant positive association between processed meat intake and hsCRP in the main analysis was diluted. In females, rice and fish intakes showed positive dose-response relationships with hsCRP, whereas poultry intake showed an inverse one. Additionally, the significant inverse association between bread intake and hsCRP in the main analysis disappeared. However, the directions of the associations were consistent between the analysis using complete data and that using multiply imputed data (Table 2 and Supplemental Table 7).
